# Cluster randomised controlled trial to examine medical mask use as source control for people with respiratory illness

**DOI:** 10.1136/bmjopen-2016-012330

**Published:** 2016-12-30

**Authors:** Chandini Raina MacIntyre, Yi Zhang, Abrar Ahmad Chughtai, Holly Seale, Daitao Zhang, Yanhui Chu, Haiyan Zhang, Bayzidur Rahman, Quanyi Wang

**Affiliations:** 1 School of Public Health and Community Medicine UNSW Medicine University of New South Wales, Sydney, New South Wales, Australia; 2 College of Public Service & Community Solutions, Arizona State University, Phoenix, Arizona, USA; 3 The Beijing Centre for Disease Prevention and Control Beijing China, XiCheng district CDC Beijing China, Dongcheng district CDC Beijing, Beijing, China

**Keywords:** Mask, Influenza

## Abstract

**Rationale:**

Medical masks are commonly used by sick individuals with influenza-like illness (ILI) to prevent spread of infections to others, but clinical efficacy data are absent.

**Objective:**

Determine whether medical mask use by sick individuals with ILI protects well contacts from related respiratory infections.

**Setting:**

6 major hospitals in 2 districts of Beijing, China.

**Design:**

Cluster randomised controlled trial.

**Participants:**

245 index cases with ILI.

**Intervention:**

Index cases with ILI were randomly allocated to medical mask (n=123) and control arms (n=122). Since 43 index cases in the control arm also used a mask during the study period, an as-treated post hoc analysis was performed by comparing outcomes among household members of index cases who used a mask (mask group) with household members of index cases who did not use a mask (no-mask group).

**Main outcome measure:**

Primary outcomes measured in household members were clinical respiratory illness, ILI and laboratory-confirmed viral respiratory infection.

**Results:**

In an intention-to-treat analysis, rates of clinical respiratory illness (relative risk (RR) 0.61, 95% CI 0.18 to 2.13), ILI (RR 0.32, 95% CI 0.03 to 3.13) and laboratory-confirmed viral infections (RR 0.97, 95% CI 0.06 to 15.54) were consistently lower in the mask arm compared with control, although not statistically significant. A post hoc comparison between the mask versus no-mask groups showed a protective effect against clinical respiratory illness, but not against ILI and laboratory-confirmed viral respiratory infections.

**Conclusions:**

The study indicates a potential benefit of medical masks for source control, but is limited by small sample size and low secondary attack rates. Larger trials are needed to confirm efficacy of medical masks as source control.

**Trial registration number:**

ACTRN12613000852752; Results.

**Strengths and limitations of this study**

- Medical masks are commonly used to prevent spread of infection from sick individuals to others; however, data on the clinical efficacy of this approach are sparse.
- A cluster-randomised control trial was conducted to examine the efficacy of medical masks as source control.
- The sample size was small and the study was underpowered to detect a statistically significant difference in outcome in the intention-to-treat analysis.
- Removal of masks in the intervention arm during meal times may have reduced efficacy and biased the results towards the null.

## Introduction

Medical masks are commonly used in healthcare settings for two main purposes: (1) by well healthcare workers (HCWs) to protect them from infections transmitted by droplet route and splash and spray of blood and body fluids; and (2) by sick individuals to prevent transmission to others (source control).^1^ ^2^ There are currently major gaps in our knowledge about the impact of masks on the transmission of respiratory infections.^3^ Most clinical trials have been focused on the protection of the well wearer, rather than on source control.^3^ Cloth and medical masks were originally developed as source control to prevent contamination of sterile sites by the wearer in operating theatres (OTs);^4^ ^5^ however, their effectiveness in preventing surgical site infections is yet to be proven.^6–8^

Although masks are also widely used in the community to prevent spread of infection from sick and infectious people,^4^ ^9–12^ the majority of data on their use are observational and derived from outbreaks and pandemics. Among the nine randomised controlled trials (RCTs) in household and community settings until now,^3^ only one examined the role of masks as source control and was inconclusive.^13^ In other clinical trials, masks were either used by both sick patients (index cases as source control) and their household members^14–16^ or only by household members.^17–19^ Most of these studies failed to show any efficacy of mask use in preventing spread of infections from the sick individuals.

Masks are also used to prevent surgical site infections in the OT,^3^ although most studies failed to show any efficacy against this indication.^6–8^ ^20^ Only one clinical trial reported high infection rates after surgery if masks were not used by the surgeon in the OT.^21^ Among the five clinical trials in the healthcare setting to test the efficacy of masks/respirators as respiratory protection,^3^ none examined the use of masks as source control. Laboratory studies generally support the use of medical masks to prevent spread of infections from patients with influenza and tuberculosis (TB) to their contacts.^22–24^

Mask use as source control in healthcare settings has now been included in standard infection control precautions during periods of increased respiratory infection activity in the community, yet there is no clinical efficacy evidence to support this recommendation. The aim of this study was to determine whether medical mask use by people in a community setting with influenza-like illness (ILI) protects well contacts from infection.

## Methods

### Design

An RCT was conducted in fever clinics in six major hospitals in two districts of Beijing, China. The fever clinics are outpatient departments for the assessment and treatment of febrile patients. The recruitment of participants was started on 18 November 2013 and completed on 20 January 2014. Adults who attended the fever clinic were screened by hospital staff to identify if they were eligible for the study. A study staff member approached eligible patients when they presented in the clinic and invited them to participate in the study. Recruited patients meeting the case definition of ILI (see below) were referred to as index cases, which was the first case in a potential chain of infection transmission.

### Eligibility

Patients aged 18 years and older (index cases) with ILI (defined as fever ≥38°C plus one respiratory symptom including cough, nasal congestion, runny nose, sore throat or sneezes) who attended a fever outpatient clinic during the study period, had no history of ILI among household members in the prior 14 days and who lived with at least two other people at home were recruited for the study. ILI was used as a selection criterion to achieve high specificity for index cases. Patients who were unable or refused to give consent, had onset of symptoms >24 hours prior to recruitment, were admitted to hospital, resided in a household with <2 other people, or had other ill household members at home were excluded from the study.

### Randomisation

**Figure 1:**
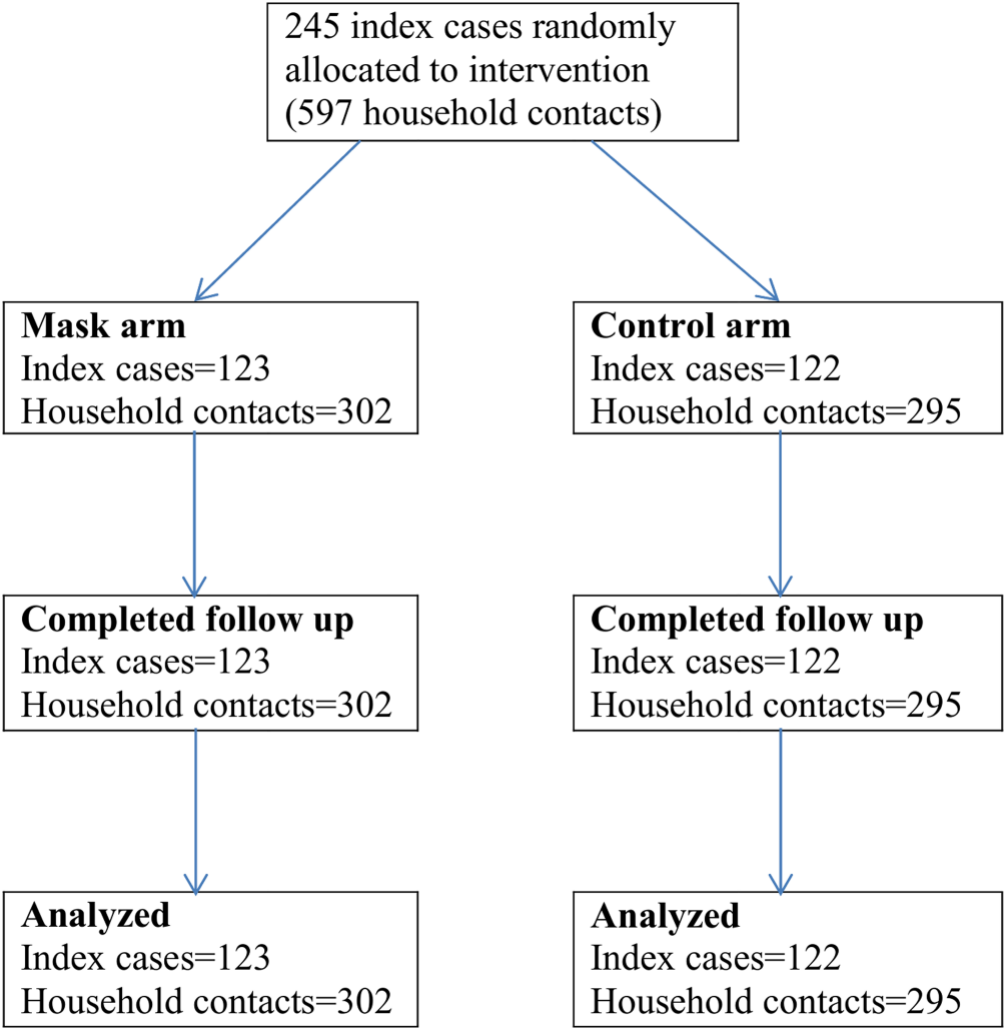
Consort diagram of recruitment and follow-up.

After providing informed consent, 245 index cases were included and randomly allocated to intervention (mask) and control (no-mask) arms. A research team member (YZ) performed the random allocation sequence using Microsoft Excel and doctors enrolled the participants randomly to intervention and control arms. Patients had an equal chance to be in the either intervention or control arm. One hundred and twenty-three index cases and 302 household contacts were included in the mask (source control) arm and 122 index cases and 295 household contacts were included in the control arm (figure 1). Cases and their household contacts were assigned together as a cluster to either the intervention or control arm.

### Intervention

The mask or no-mask intervention was applied to the index cases and respiratory illness was measured in household contacts. Index cases (patients with ILI) in the intervention arm wore a medical mask at home. Index cases were asked to wear a mask (3M 1817 surgical mask) whenever they were in the same room as a household member or a visitor to the household. They were allowed to remove their masks during meal times and while asleep. Index cases were shown how to wear the mask and instructed to wash their hands when donning and doffing the mask. Index cases were provided with 3 masks per day for 7 days (21 masks in total). They were informed that they could cease wearing a mask once their symptoms resolved. Index cases in the control arm did not receive any intervention. Mask use by other household members was not required and not reported.

### Outcome measures

Respiratory illness outcomes were measured in household contacts of the index cases. Primary end points measured in household contacts included: (1) clinical respiratory illness (CRI), defined as two or more respiratory symptoms (cough, nasal congestion, runny nose, sore throat or sneezes) or one respiratory symptom and a systemic symptom (chill, lethargy, loss of appetite, abdominal pain, muscle or joint aches); (2) ILI, defined as fever ≥38°C plus one respiratory symptom; and (3) laboratory-confirmed viral respiratory infection, defined as detection of adenoviruses, human metapneumovirus, coronaviruses 229E/NL63 and OC43/HKU1, parainfluenzaviruses 1, 2 and 3, influenza viruses A and B, respiratory syncytial virus A and B, or rhinovirus A/B by nucleic acid testing (NAT) using a commercial multiplex PCR (Seegen, Seoul, Korea).^25–27^

If any respiratory or systemic symptoms occurred in household members, index cases were instructed to notify the study coordinator. Symptomatic household members were asked to complete ‘sick follow-up’ questionnaires and anyone who met the CRI definition was tested for laboratory-confirmed viral respiratory infections.

### Data collection and follow-up

At baseline, detailed clinical and demographic information including household structure was collected from index cases and their household members. This included age, sex, smoking history, comorbidities, medications, hand washing practices, influenza vaccination and normal practices around the use of masks.

Follow-up period (7 days): Each index case was asked to keep a diary to record activities, symptoms and daily temperatures for 7 days. Symptoms in the household members were also recorded in the diary cards and index cases were asked to report any symptom. The index cases were asked to contact the study coordinator if any of the following symptoms appeared in household members: cough, nasal congestion, runny nose, sore throat, sneezes, chill, lethargy, loss of appetite, abdominal pain and muscle or joint aches. The study coordinator then assessed the household member and completed a follow-up survey. Samples were obtained from all symptomatic cases. All index cases in the intervention and control arms were also asked to document compliance with mask use.^26^ ^27^ Diary cards to record mask use were given to each index case, and they were asked to carry them during the day. Diary cards were returned to the investigators at the end of the study. The study coordinator also contacted index cases via telephone on every alternate day to check whether any household member developed symptoms. Assessors were not blinded, because the intervention (mask wearing) was visible. However, laboratory testing was blinded.

### Sample collection and laboratory testing

Samples were collected from index patients at the time of recruitment and from symptomatic household members during follow-up. Household members were provided with an information sheet and written consent was sought before sampling. Only those household members who provided consent were swabbed. If the sick household member was aged <18 years, consent was obtained from a parent or guardian. Swabs were taken at the home by trained investigators.

Double rayon-tipped, plastic-shafted swabs were used to swab both tonsillar areas and the posterior pharyngeal wall of symptomatic participants. The swabs were then transported immediately after collection to the Beijing Centre for Disease Control (CDC) laboratories, or stored at 4°C within 48 hours if transport was delayed.

Viral DNA/RNA was extracted from each respiratory specimen using the Viral Gene-spin TM Kit (iNtRON Biotechnology, Seoul, Korea) according to the manufacturer's instructions. Reverse transcription was performed using the RevertAidTM First Strand cDNA Synthesis Kit (Fermentas, Ontario, Canada) to synthesise cDNA. Multiplex PCR was carried out using the Seeplex RV12 Detection Kit (Seegen, Seoul, Korea) to detect adenoviruses, human metapneumovirus, coronavirus 229E/NL63 and OC43/HKU1, parainfluenzaviruses 1, 2 or 3, influenza viruses A or B, respiratory syncytial virus A or B, and rhinovirus A/B. A mixture of 12 viral clones was used as a positive control template, and sterile deionised water was used as a negative control. Viral isolation by Madin Darby Canine Kidney (MDCK) cell culture was undertaken for some of the influenza samples that were NAT positive. Specimen processing, DNA/RNA extraction, PCR amplification and PCR product analyses were conducted in different rooms to avoid cross-contamination.

### Sample size

In this cluster-randomised design, the household was the unit of randomisation and the average household size was three people. Assuming that the attack rate of CRI in the control households was 16–20% (based on the results of a previously published household mask trial),^17^ with a 5% significance level and 85% power and a minimum relative risk (RR) of 0.5 (intervention/control), 385 participants were required in each arm, which was composed of 118 households and, on average, 3 members per household. In this calculation, we assumed that the intracluster correlation coefficient (ICC) was 0.1. An estimated 250 patients with ILI were recruited into the study to allow for possible index case dropout during the study.

### Data analysis

Descriptive statistics were compared in the mask and control arms and respiratory virus infection attack rates were quantified. Data from the diary cards were used to calculate person-days of infection incidence. Primary end points were analysed by intention to treat across the study arms and ICC for clustering by household was estimated using the clchi2 command in Stata.^28^ RRs were calculated for the mask arm. The Kaplan-Meier survival curves were generated to compare the survival pattern of outcomes across the mask and control arms. Differences between the survival curves were assessed through the log-rank test. The analyses were conducted at the individual level and HRs were calculated using the Cox proportional hazards model after adjusting for clustering by household by adding a shared frailty to the model. Owing to the very few outcome events encountered, a multivariable Cox model was not appropriate. We checked the effect of individual potential confounders on the outcome variable fitting univariable Cox models. Since there were 10 cases of CRI, we included this variable in a multivariable cluster-adjusted Cox model. Multivariate analyses were not performed for ILI and laboratory-confirmed viruses because of low numbers.

A total of 43 index cases in the control arm also used a mask during the study period (at least 1 hour per day) and 7 index cases in the masks arm did not use a mask at all, so a post hoc sensitivity analysis was carried out to compare outcomes among household members of index cases who used a mask (hereafter ‘mask group’) with those of index cases who did not use a mask (hereafter ‘no-mask group’). All statistical analyses were conducted using Stata V.13 (StataCorp. Stata 12 base reference manual. College Station, Texas, USA: Stata Press, 2011).

## Results

A total of 245 index patients were randomised into the mask arm (n=123) or the control arm (n=122). The mask arm had on average 2.5 household contacts per index case (n=302), while the control arm had 2.4 household contacts per index case (n=295). Characteristics of index cases and household members are presented in table 1. There was no significant difference between arms, and most characteristics, including medication use (data not shown), were generally similar. Viruses were isolated from 60% (146/245) of index cases. Influenza was the most common virus isolated from 115 (47%) cases—influenza A—100, influenza B—11 and influenza A and B—4. Other viruses isolated from index cases were rhinovirus,^13^ NL63^11^ and C229E.^7^ More than one virus was isolated in 48 (20%) index cases, including 17 coinfections with influenza.

**Table 1:** Demographic and other characteristics of the index cases and household members

Variable	Mask arm (% and 95% CI)	Control arm (% and 95% CI)
Index case (number)	123	122
Gender (male)	56/123 45.5% (37.0% to 54.3%)	45/122 36.9% (28.8% to 45.7%)
Age (mean)	40.2 (37.6 to 42.8)	39.7 (37.3 to 42.0)
Education (undergraduate/postgraduate)	78/123 63.4% (54.6% to 71.4%)	74/122 60.7% (51.8% to 68.9%)
Smoker (current/ex)	29/123 23.6% (16.9% to 31.8%)	26/122 21.3% (15.0% to 29.4%)
Pre-existing illness*	21/123 17.1% (16.2% to 31.0%)	16/122 13.1% (8.2% to 20.2%)
Influenza vaccination (yes)	5/123 4.1% (1.7% to 9.2%)	5/122 4.1% (1.8% to 9.2%)
Hand washing (most/all times)	98/123 79.7% (71.7% to 85.8%)	109/122 89.3% (82.6% to 93.7%)
Average hour of home stay†	16.6 (15.9 to 17.3)	16.6 (15.9 to 17.3)
Average hour mask wearing†	4.4 (3.9 to 4.9)	1.4 (0.9 to 1.8)
Household (members)	302	295
Number of household per arm	2.5	2.4
Gender (male)	149/302 49.3% (43.4% to 24.6%)	168/295 56.9% (51.6% to 62.9%)
Influenza vaccination (yes)‡	22/298 7.4% (4.9% to 10.9%)	30/285 10.5% (7.1% to 14.6%)
Age (mean)	38.3 (36.0 to 40.5)	36.4 (34.1 to 38.8)

*Includes asthma, chronic obstructive pulmonary disease, diabetes, ischaemic heart disease, immune-compromised and others.

†Variable was created by taking average hours over the trial period.

‡Missing data for 14 cases.

**Figure 2:**
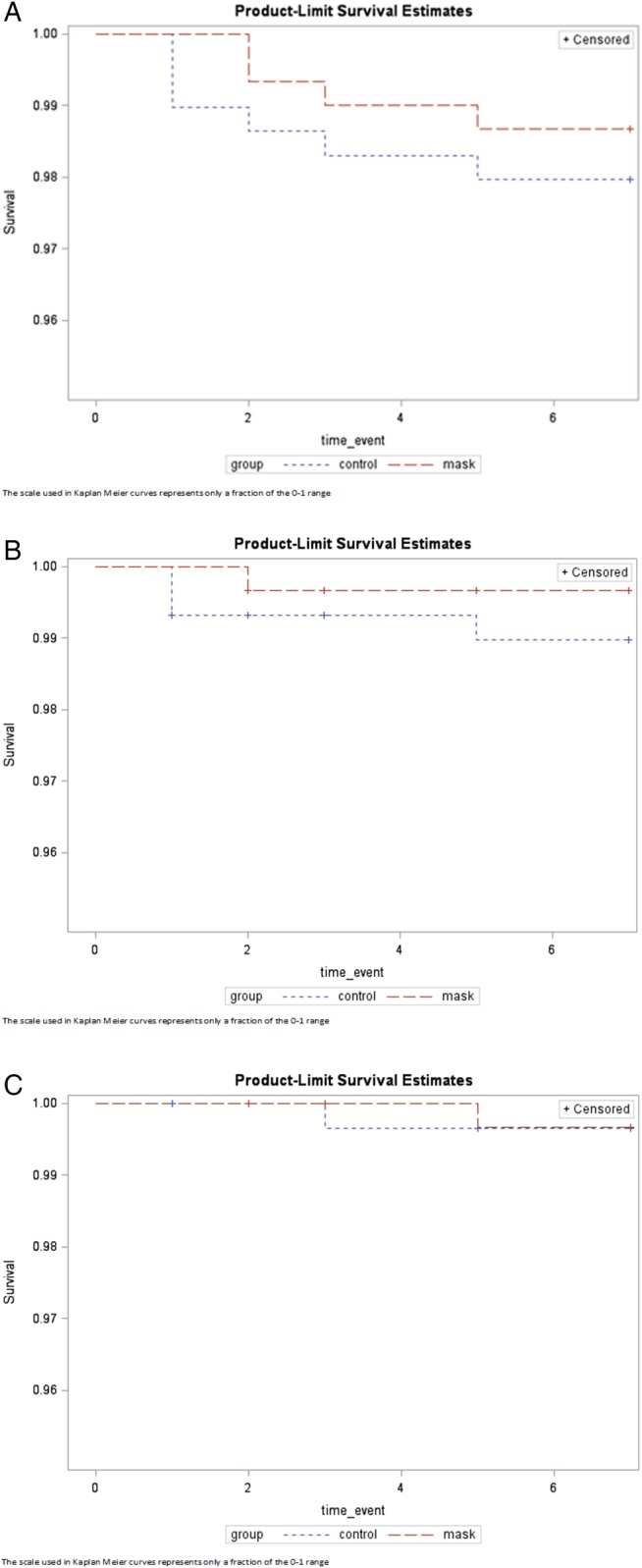
Survival curves for medical mask versus control arms (2A–C). The scale used in Kaplan Meier curves represents only a fraction of the 0–1 range.

Table 2 shows the intention-to-treat analysis. CRI was reported in four (1.91/1000 person-days) household members in the mask arm, compared with six household members (2.95/1000 person-days) in the control arm (RR 0.65, 95% CI 0.18 to 2.29). Only one case (0.48/1000 person-days) of ILI was reported in the mask arm, compared with three cases (1.47/1000 person-days) in the control arm (RR 0.32, 95% CI 0.03 to 3.11). Two laboratory-confirmed infections were identified among symptomatic household members from a separate household. One household member had the same infection (influenza H1N1) as the respective index case. Rhinovirus was isolated from another household member. However, no pathogen was isolated from the respective index case. The two cases of laboratory-confirmed viral respiratory infections of household members occurred in separate study arms (RR 0.97, 95% CI 0.06 to 15.5). The Kaplan-Meier curves showed no significant differences in the outcomes between two arms (p>0.050; figure 2).

**Table 2:** Number and proportion of household members reporting primary outcomes, by randomisation arm and intention-to-treat analysis (n=597)*

	CRI No (rate person-days)	RR (95% CI)	ILI No (rate person-days)		Laboratory-confirmed viral respiratory infections No (rate person-days)	RR (95% CI)
Mask arm†	4/2098 (1.91/1000)	0.65 (0.18 to 2.29)	1/2098 (0.48/1000)	0.32 (0.03 to 3.11)	1/2098 (0.48/1000)	0.97 (0.06 to 15.5)
Control arm‡	6/2036 (2.95/1000)	Ref	3/2036 (1.47/1000)	Ref	1/2036 (0.49/1000)	Ref

*Household members (mask arm 302 and control arm 295).

†Intracluster correlation coefficient is <0.001.

‡Intracluster correlation coefficient is <0.001.

CRI, clinical respiratory illness; ILI, influenza-like illness; RR, relative risk.

The duration of contact of index cases with household members was 10.4 and 11.1 hours in the mask and control arms, respectively. On average, participants in the mask arm used a mask for 4.4 hours, while participants in the control arm used a mask for 1.4 hours. In a univariable Cox model, only the age of the household contact was significantly associated with the CRI (table 3). There was no association between mask use by the index cases and rates of infectious outcomes in household members (table 3). Although the risks of CRI (RR 0.61, 95% CI 0.18 to 2.13), ILI (RR 0.32, 95% CI 0.03 to 3.13) and laboratory-confirmed viral infections (RR 0.97, 95% CI 0.06 to 15.54) were lower in the mask arm, the difference was not statistically significant.

**Table 3:** HRs from shared frailty Cox proportional hazards model for household members in masks versus control arms (n=597)*

	CRI HR (95% CI)†	ILI HR (95% CI)†	Laboratory-confirmed viral respiratory infections HR (95% CI)†
Masks arm (index case)	0.61 (0.18 to 2.13)	0.32 (0.03 to 3.13)	0.97 (0.06 to 15.54)
Control arm (index case)	Ref	Ref	Ref
Age (household)	1.03 (1.01 to 1.05)		

*Household members (mask arm 302 and control arm 295).

†Multivariate analysis was performed as there were 10 cases of CRI and age was also significant in the univariate analysis. Multivariate analyses were not performed for ILI and laboratory-confirmed viral respiratory infections due to the low number of cases.

CRI, clinical respiratory illness; ILI, influenza-like illness.

**Figure 3:**
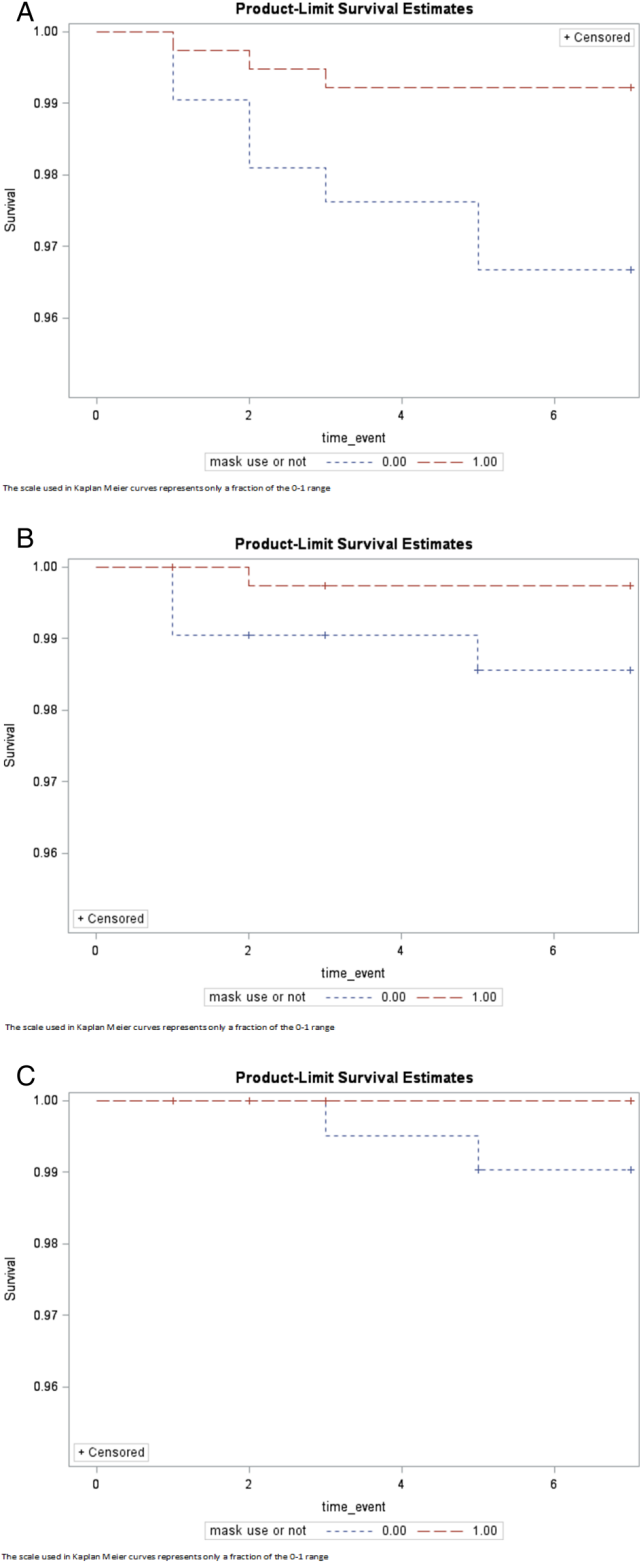
Survival curves for mask versus no-mask group (3A–C). The scale used in Kaplan Meier curves represents only a fraction of the 0–1 range.

Tables 4 and 5 show a sensitivity analysis comparing outcomes among household members of index cases using a mask (mask group) with those of index cases who did not use a mask (no-mask group). Overall, 159 index cases (65%) used a mask during the trial period including 43 participants from the control arm. Three hundred and eighty-seven household members were included in the mask group and 210 were included in the no-mask group. Rates of all outcomes were lower in the mask group, and CRI was significantly lower in the contacts of the mask group compared with the contacts of the no-mask group. The Kaplan-Meier curves (figure 3) showed a significant difference in the rate of CRI among the mask and no-mask groups (p 0.020).

**Table 4:** Number and proportion of participants reporting primary outcomes, by mask versus no-mask groups (n=597)*

	CRI No (rate person-days)	RR	ILI No (rate person-days)	RR	Laboratory-confirmed viral respiratory infections No (rate person-days)	HR†
Mask group	3/2694 (1.11/1000)	0.23 (0.06 to 0.88)	1/2694 (0.37/1000)	0.18 (0.02 to 1.71)	0/2694 (0/1000)	0.11 (0.01 to 4.40)
No-mask group	7/1440 (4.86/1000)	Ref	3/1440 (2.08/1000)	Ref	2/1440 (0.70/1000)	Ref

*Household members (mask group 387 and no-mask group 210).

†Calculated through Cox PH methods.

CRI, clinical respiratory illness; ILI, influenza-like illness; PH, proportional hazards; RR, relative risk.

**Table 5:** HRs from shared frailty Cox proportional hazards model for mask versus no-mask groups (no randomization; n=597)*

	CRI HR (95% CI)†	ILI HR (95% CI)†	Laboratory-confirmed viral respiratory infections HR (95% CI)†
Masks group (index case)	**0.22 (0.06 to 0.86)**	0.18 (0.02 to 1.73)	0.11 (0.01 to 4.40)
No-mask group (index case)	Ref	Ref	Ref
Age (household)	1.03 (1.00 to 1.06)		

Bold values are statistically significant results.

*Household members (mask group 387 and no-mask group 210).

†Multivariate analysis was performed as there were 10 cases of CRI and age was also significant in the univariate analysis. Multivariate analyses were not performed for ILI and laboratory-confirmed viral respiratory infections due to the low number of cases.

CRI, clinical respiratory illness; ILI, influenza-like illness.

After adjusting for the age of household contacts, the risk of CRI was 78% lower in the contacts of the mask group (RR 0.22, 95% CI 0.06 to 0.86), compared with contacts of the no-mask group. Although the risks of ILI (RR 0.18, 95% CI 0.02 to 1.73) and laboratory-confirmed viral respiratory infections (RR 0.11, 95% CI 0.01 to 4.40) were also lower in the mask group, the difference was not statistically significant.

## Discussion

Masks are commonly recommended as source control for patients with respiratory infections to prevent the spread of infection to others,^2^ ^3^ but data on the clinical efficacy of this approach are sparse. We did not find a significant benefit of medical masks as source control, but rates of CRI and ILI in household members were consistently lower in the mask arm compared with the control arm. The study was underpowered to detect a statistically significant difference. The additional analysis by actual mask use showed significantly lower rates of CRI in the mask group compared with the no-mask group, suggesting that larger trials should be conducted to further examine the efficacy of masks as source control.

Our findings are consistent with previous research in community and household settings, where the efficacy of masks as source control was measured. Until now, only one RCT has been conducted in the community setting to examine the role of masks in preventing spread of infection from wearers.^3^ Canini and colleagues conducted an RCT in France during the 2008–2009 influenza season and randomised index patients into medical mask (52 households and 148 contacts) and control arms (53 households and 158 contacts). ILI was reported in 16.2% and 15.8% of contacts in the intervention and control arms, respectively, and the difference was not statistically significant (mean difference 0.40%, 95% CI −10% to 11%, p=1.00). The trial was concluded early due to low recruitment and the subsequent influenza A (H1N1)pdm09 pandemic.^13^ In addition, masks were also used by index cases and household members in some community-based RCTs with mixed interventions.^14^ ^15^ Cowling and colleagues conducted two RCTs in Hong Kong to examine the efficacy of masks, and index cases were randomised into medical mask, medical mask plus hand hygiene, hand hygiene and control arms. Both index cases and household members used masks. The rates of laboratory-confirmed influenza and ILI were the same in the intervention and control groups in the intention-to-treat analysis.^14^ However, in the second trial, mask use with hand hygiene was protective in household contacts when the intervention was applied within 36 hours of onset of symptoms in the index case (OR 0.33, 95% CI 0.13 to 0.87).^15^ Since masks were used by sick patients and their household members in these studies, the effect of mask being ‘source control’ is more difficult to quantify precisely.

Masks are not designed for respiratory protection and are commonly used in the healthcare setting to prevent spread of infections from the wearer, whether worn by a sick patient or well staff member.^1^ ^3^ One such use is the wearing of masks by well surgeons and other OT staff to protect patients from contamination during surgery. Presumably, the exhaled pathogen load would be much higher in a sick patient compared with a well surgeon, and therefore the use of a mask for source control in sick patients may have more benefit than OT use of source control.

This study has some limitations. The sample size was small and the study may have been underpowered to detect a statistically significant difference in outcome in the intention-to-treat analysis. Post hoc analysis, however, showed a potential benefit of medical masks for source control. It is possible that infection transmission may have occurred during meal times (when patients were not required to wear a mask). This would have the effect of biasing the results towards the null. In the sample size calculations, we assumed a 16–20% attack rate of CRI in the control arm, based on the results of a previously published household mask trial.^17^ However, the secondary attack rates were much lower in this study which might be due to testing only symptomatic cases.

In a univariable Cox model, only the age of household contact was significantly associated with the CRI. All other variables were uniformly distributed among the study arms, so we only adjusted for the age of the household contact in the analysis of CRI as an outcome. Multivariate analyses were not performed for ILI and laboratory-confirmed viruses. However, some variables may have an impact on the number of events. For example, the rates of hand hygiene were higher among the ‘control’ arm compared with the mask arm (109/122, 89.3% vs 98/123, 79.7%), which may have had an impact on the number of outcome events. Owing to the low event rates and non-significant difference of hand hygiene among the two arms, we did not adjust for hand hygiene in any analysis. Further, inclusion of hand hygiene in the model did not change the HR. Finally, post hoc analyses are potentially biased due to loss of randomisation and it was added as a sensitivity analysis in this study because of deviations from protocol in mask wearing.

Despite a lack of evidence, most health organisations and countries recommend the use of masks by sick patients as source control.^1^ ^2^ Masks are used commonly by patients with TB, although clinical trials have not been conducted for this indication. There is a need to conduct larger trials to confirm the suggestion of benefit in our study. If source control is effective in reducing hospital transmission of infection, this may have a practical benefit to mitigate the problem of poor compliance with mask wearing among well HCWs.^3^ Compliance with any intervention for someone who is well and asymptomatic is far more challenging than compliance in people who are unwell,^29^ so source control may have an important role in hospital infection control. Reducing the transmission of respiratory pathogens by source patients could also have further benefits in the community in preventing transmission of infection to close contacts such as those in the same household, and should be studied further.
